# Integrative Biology of *Idas iwaotakii* (Habe, 1958), a ‘Model Species’ Associated with Sunken Organic Substrates

**DOI:** 10.1371/journal.pone.0069680

**Published:** 2013-07-24

**Authors:** Justine Thubaut, Laure Corbari, Olivier Gros, Sébastien Duperron, Arnaud Couloux, Sarah Samadi

**Affiliations:** 1 Département Systématique et Evolution, UMR 7138 UPMC-IRD-MNHN-CNRS, Muséum national d’Histoire naturelle, Paris, France; 2 Département de Biologie, UMR-CNRS 7138, Université des Antilles et de la Guyane, Point-à-Pitre, Guadeloupe, France; 3 Adaptation aux milieux extrêmes, UMR 7138 UPMC-IRD-MNHN-CNRS, Université Pierre et Marie Curie, Paris, France; 4 Genoscope, Centre National de Séquençage, Evry, France; Australian Museum, Australia

## Abstract

The giant bathymodioline mussels from vents have been studied as models to understand the adaptation of organisms to deep-sea chemosynthetic environments. These mussels are closely related to minute mussels associated to organic remains decaying on the deep-sea floor. Whereas biological data accumulate for the giant mussels, the small mussels remain poorly studied. Despite this lack of data for species living on organic remains it has been hypothesized that during evolution, contrary to their relatives from vents or seeps, they did not acquire highly specialized biological features. We aim at testing this hypothesis by providing new biological data for species associated with organic falls. Within Bathymodiolinae a close phylogenetic relationship was revealed between the *Bathymodiolus sensu stricto* lineage (i.e. *“thermophilus”* lineage) which includes exclusively vent and seep species, and a diversified lineage of small mussels, attributed to the genus *Idas*, that includes mostly species from organic falls. We selected *Idas iwaotakii* (Habe, 1958) from this latter lineage to analyse population structure and to document biological features. Mitochondrial and nuclear markers reveal a north-south genetic structure at an oceanic scale in the Western Pacific but no structure was revealed at a regional scale or as correlated with the kind of substrate or depth. The morphology of larval shells suggests substantial dispersal abilities. Nutritional features were assessed by examining bacterial diversity coupled by a microscopic analysis of the digestive tract. Molecular data demonstrated the presence of sulphur-oxidizing bacteria resembling those identified in other Bathymodiolinae. In contrast with most *Bathymodiolus s.s.* species the digestive tract of *I. iwaotakii* is not reduced. Combining data from literature with the present data shows that most of the important biological features are shared between *Bathymodiolus s.s.* species and its sister-lineage. However *Bathymodiolus s.s.* species are ecologically more restricted and also display a lower species richness than *Idas* species.

## Introduction

**Figure 1:**
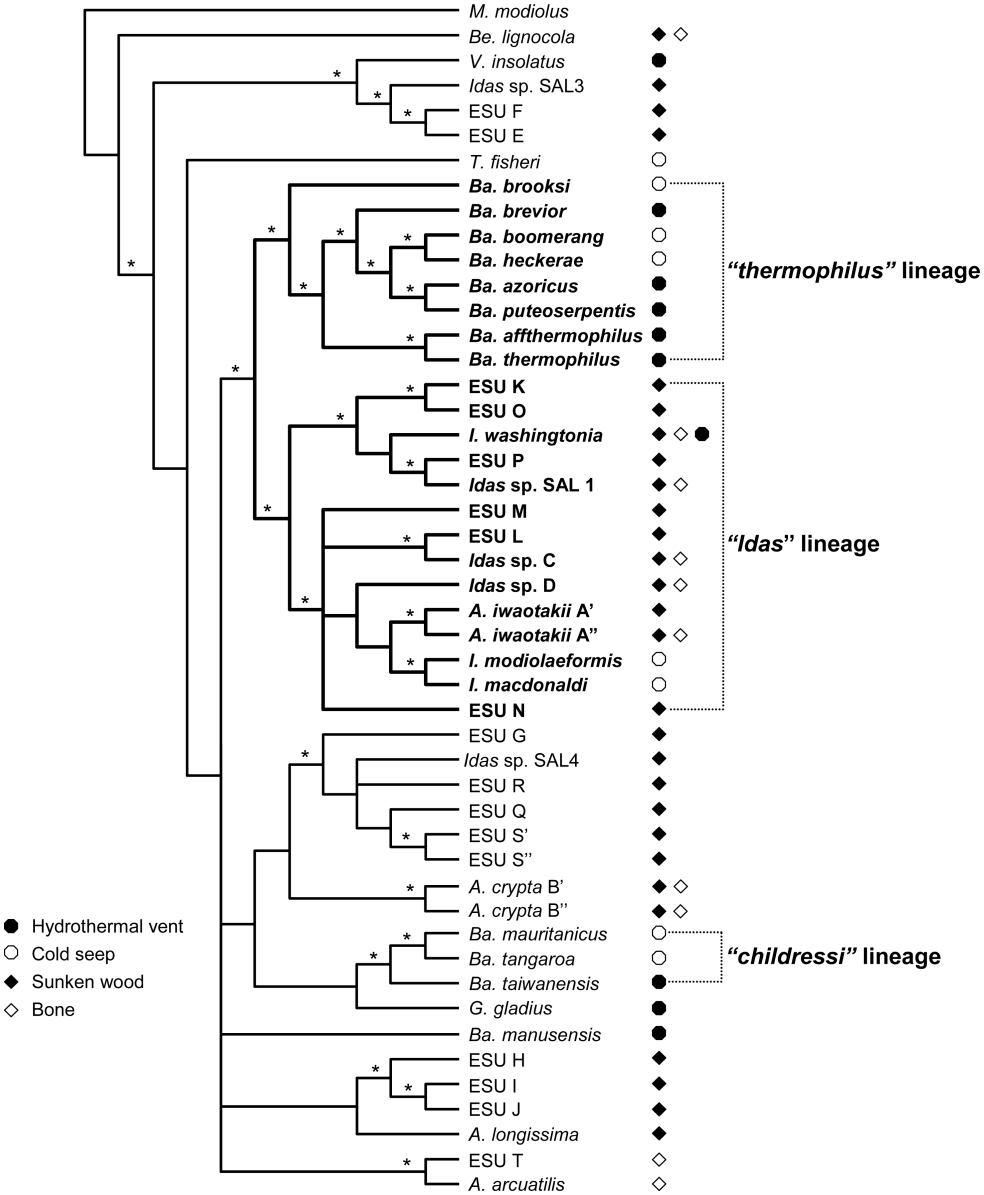
Dendrogram obtained from Bayesian analyses of the combined dataset of COI mtDNA and the 28S rRNA. Asterisks correspond to nodes with posterior probabilities (PP) obtained from BA analysis and bootstrap values obtained from ML analysis that are higher than 0.90 and 50%, respectively. (modified from [7]).

Despite the number and variety of studies on organisms from deep-sea chemosynthetic environments, their evolutionary origins and the biological processes explaining their diversity remain poorly resolved [1]. Among these organisms, bathymodioline mussels are studied as a model to elucidate the biological processes in vent and seep environments often designated as ‘extreme’ (e.g. [2], [3]). A comparative approach with the most closely related organisms from different environments is, however, required to understand the evolutionary significance of the organisms’ features in such ‘extreme’ environments. Distel et al. (2000) [4] were the first to show that bathymodioline mussels inhabiting vents and seeps form a monophyletic lineage with the modioline mussels associated with sunken organic substrates. This pioneering phylogenetic result implied the inclusion of the small and poorly-known deep-sea mussels from organic falls in the Bathymodiolinae. Recently, an evolutionary scenario was proposed in which sunken-wood is considered as an “early stage” towards adaptation to more extreme chemosynthetic environments in the deep-sea [5], [6]. However, a recent molecular phylogenetic study, including more than 20 potentially new species associated with organic remains, suggested a more complex evolutionary scenario in which the history of hydrothermal vent and cold seep species is entangled with that of the small mussels associated with organic remains (Fig. 1) [7]. Lorion et al. (2010) [7] confirmed that the vent and seep mussels are divided into several distinct lineages, among which the *“thermophilus”* group (i.e. *Bathymodiolus sensus stricto* since *thermophilus* is the type-species of the genus) is the most thoroughly-studied. This clade is closely related to a much more diversified lineage of small mussels, here provisionally attributed to the genus *Idas*, with most of the species associated with organic remains but also with a few from vents or seeps (Fig. 1). Unfortunately, biological data are scarce for most species within this lineage [7] and thus the evolutionary significance of biological features observed in vent and seep species is difficult to establish.

One of the most striking feature discovered with these ‘extreme’ environments is symbiotic interactions with chemosynthetic bacteria [8], interpreted as a key adaptation of bathymodioline mussels to the particular environmental conditions of deep-sea vents and seeps. In *Bathymodiolus s.s.,* chemosynthetic bacteria use reduced compounds such as hydrogen sulphide (H_2_S) and/or methane (CH_4_) to gain energy and for carbon fixation from dissolved CO_2_ or methane providing a source of carbon to their host [8]. Most species within *Bathymodiolus s.s.* have both sulphur- and methane-oxidising symbionts, (e.g. [9]). Hydrogen was also recently shown to be an additional energy source for the sulphur-oxidising symbionts of *Bathymodiolus* (s.s.) *puteoserpentis* [10]. In all species, bacteria are located within the gill epithelial cells suggesting a tight relationship between metazoan and bacterial partners. Moreover for several *Bathymodiolus s.s.* species, the digestive tract is reduced [11]–[15] and it has been hypothesized that filter-feeding plays only a minor role in the diet compared to the food supply offered by their symbionts, especially in deeper-dwelling species.

By contrast, for the small species within the sister lineage of *Bathymodiolus s.s*, data about symbiosis are only available for two cold seep species (*Idas modiolaeformis*: [16], [17]; *I. macdonaldi*: [9]) and for three species associated with organic remains (*Idas washingtonius* on whale bone: [18]–[20]; *Idas* sp. D on wood falls: [8] and *Idas* sp. C on both wood and bone: [21]). Located on gills, symbiotic bacteria are intracellular in *I. washingtonius* and *I. macdonaldi* but extracellular for *I.* sp. D and *I.* sp. C. Up to six different bacteria were observed in the gills of the seep species *I. modiolaeformis* [16], [17], meanwhile other species seem to harbour only sulphur-oxidisers. No data are available concerning the anatomy of the digestive tracts of *Idas* species.

Larval dispersal is a key issue for understanding the evolutionary success of specialized species, because hydrothermal vents and cold seeps are patchily distributed over large distances [22]. In bivalves, larval dispersal capability can be inferred, to a certain extent, from larval shell morphology. Indeed since the size of the prodissoconch I and of the oocyte are correlated, the size of the prodissosconch II relative to that of the prodisoconch I is indicative of the time spent in plankton by larvae [23]. Most bathymodioline species are considered planktotrophic because of their small egg size and prodissoconch morphology. Moreover, evidence for long-distance dispersal is available for several *Bathymodiolus s.s.* species [24]–[27]. The distribution of organic falls is patchy, but accumulations of vegetal remains are predictably found in the vicinity of tropical islands [28]. Several species associated with wood falls can also prosper on animal remains [7]. Thus, animal remains (notably the carcasses of large marine vertebrates), although not predictably located, are spread throughout the sea floor [29] and may constitute steps for dispersal among distant locations.

No study has yet documented population connectivity patterns among mussels associated with organic remains. We selected the species *Idas iwaotakii* to initiate such studies. This species was frequently sampled during the cruises of the Tropical Deep-sea Benthos program [28]. It has been found mainly on wood susbtrates, but also occurs on turtle bones [7]. It is distributed throughout the south-western Pacific. *I. iwaotakii* is divided into two genetic lineages [7], [30]. While the first has only been found in Japan and the Philippines, the second was found throughout the Western Pacific (sampled in Japan, Vanuatu and New Caledonia).

To document connectivity patterns in *I. iwaotakii*, we describe larval development and morphology of prodissoconchs and infer the genetic structure of populations at different geographic scales considering two environmental factors (depth and organic substrate type). We describe the bacteria associated with the gill tissue and describe the structure of the digestive tract to infer the nature of its diet. We also re-evaluate the evolutionary significance of features often presented as adaptation to extreme environments by comparing the biological features revealed here with those available for other species within the same clade (*I. modiolaeformis*, *I. macdonaldi* and *I. washingtonius*) or for species of *Bathymodiolus s.s*.

## Materials and Methods

### Sampling

**Figure 2:**
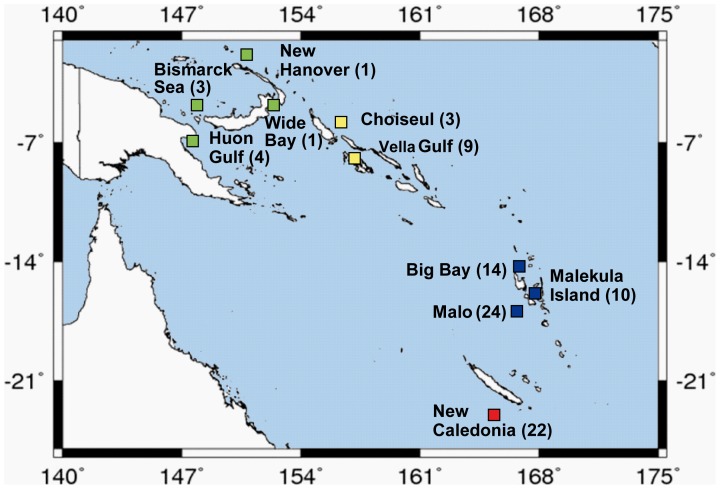
South Western Pacific map showing the sampling localities of mussels of *Idas iwaotakii*. Specimens from Malo and New Caledonia have already been studied by [7], [30].

Specimens were collected during recent cruises of the Tropical Deep-sea Benthos program [31] off the Solomon Islands (2004), Vanuatu (2005, 2006), the Philippines (2005, 2007), New Caledonia (2008) and Papua New Guinea (2010) [28]. Part of the material has already been used as described in Lorion et al. (2009, 2010) [7], [30]. The studied organisms are not protected and do not require specific export permits. Research permits were obtained for the EEZ of each country. In the Solomon Islands and Vanuatu, officers from Fisheries departments collaborated on the cruises. In the Philippines and Papua New Guinea, cruises were organised under a Memorandum of Understanding with Bureau of Fisheries and Aquatic Resources and the University of Papua New Guinea respectively. Collecting in New Caledonia was done within the French EEZ with a French vessel and no permit was required at the time of the cruises. Mussels were collected by trawling and dredging between 460 m and 2,307 m depth. Sampling locations include the Philippines (7 specimens), Papua-New Guinea (9 specimens), the Solomon Islands (12 specimens), Vanuatu (48 specimens) and New Caledonia (22 specimens) (Table 1 and Fig. 2). Specimens were preserved in 80% EtOH and are deposited in the collections of the Muséum National d’Histoire Naturelle (MNHN).

**Table 1:** Geographical localities, substrates, depths and sequences of specimens of *I. iwaotakii* used in this study.

Locality	Latitude	Longitude	Substrate	Depth (m)	COI	28S	16S bac
Japan	unknown		wood	490	(3) [^31^]		
Japan	unknown		wood	490	(3) [^7^]	(2)*	
Philippines	9°24.3′N	124°10.70′ E	wood	1764	(1) [^30^]	(1) [^30^]	
Philippines	9°25.60′N	124°2.10′E	wood	1750-1767	(1)*	(1)*	(7)*
Philippines	9°20.90′N	124°8.70′E	wood	1764	(3)*	(3)*	(10)*
Philippines	8°51.0′N	123°10.0′E	wood	982–989	(1)*	(1)*	
Philippines	8°51.30′N	122°58.90′E	wood	2303−2307	(1)*	(1)*	
Philippines	unknown		wood	unknown	(1)*		
Papua-New Guinea	6°55.85′S	147°08.22′E	wood	700−740	(2)*	(1)*	
Papua-New Guinea	6°46.98′S	147°12.53′E	wood	592−660	(2)*		
Papua-New Guinea	2°14.0′S	150°15.10′E	wood	490−505	(1)*	(1)*	
Papua-New Guinea	5°04.24′S	152°0.21′E	wood	752−998	(3)*	(2)*	
Papua-New Guinea	5°04.09′S	152°02.78′E	wood	782−1085	(1)*	(1)*	
Solomon Islands	6°37.60′S	156°13.15′E	wood	490−520	(1)*		
Solomon Islands	6°38.27′S	156°13.20′E	wood	508−522	(2)*		
Solomon Islands	7°44.50′S	156°27.90′E	wood	518−527	(1)*	(1)*	
Solomon Islands	7°42.83′S	156°24.45′E	wood	686−690	(1)*	(1)*	
Solomon Islands	7°45.21′S	156°25.61′E	wood	650−673	(2)*	(2)*	
Solomon Islands	7°57.50′S	156°51.35′E	wood	460−487	(3)*		
Solomon Islands	7°54.35′S	156°50.86′E	wood	515−520	(2)*		
Vanuatu	15°42.50′S	167°03.00′E	wood	441	(24) [^30^]	(13) [^30^] (3)*	(9)* (3)*
Vanuatu	15°3.08′S	166°56.10′E	wood	548−560	(1)*		
Vanuatu	15°1.41′S	166°53.76′E	wood	630−670	(11)*	(4)*	
Vanuatu	15°1.42′S	166°53.76′E	wood	630−705	(2)*	(2)*	
Vanuatu	16°27.48′S	167°54.78′E	wood	562−580	(2)*	(1)*	
Vanuatu	16°2.67′S	167°30.35′E	wood	802−900	(3)*	(1)*	
Vanuatu	16°37.48′S	167°57.53′E	wood	641−677	(4)*	(1)*	
Vanuatu	16°27.62′S	167°53.66′E	wood	568−591	(1)*		
New Caledonia	22°33.25′ S	166°24.70′E	bone	800	(22) [^7^]	(4)*	(15)* (6)*

The number of sequences is indicated in brackets. The superscripts refer to the authors of sequences. The stars indicate the sequences obtained during this study.

### Microscopy

#### Larval shell observation

The prodissoconchs of 15 specimens (sample ID: MNHN-IM-2009–22278 to MNHN-IM-2009–22292) from New Caledonia were coated with gold-palladium and examined under a JEOL JSM-840A scanning electron microscope (SEM) operating at 80 kV. Shells were subsequently measured and described.

#### Symbiotic bacteria

The specimen used to study the localisation of symbiotic bacteria was collected off Vanuatu in association with coconut fibers. Gills were pre-fixed at sea in cacodylate-buffered 2% glutaraldehyde for two hours at room temperature (RT) and briefly rinsed in a fresh volume of the same buffer. Gills were stored at RT in that buffer until they were brought to the laboratory a few weeks later. Samples were then fixed for 45 minutes at RT in 1% osmium tetroxide in the same buffer, rinsed in distilled water and post-fixed with 2% aqueous uranyl acetate for one hour before embedding in Epon-Araldite by the method of Mollenhauer described by Glauert (1975) [32].

#### Digestive system

Two specimens (sample ID: MNHN-IM-2009–22293 and MNHN-IM-2009–22294, preservation in 80% ethanol), were chosen for the analysis of the digestive tract structure, both living on turtle bone off New Caledonia. Digestive tracts of specimens (n = 2) were observed in longitudinal sections, and cross sections. Mussels were removed from their shell and whole individuals were first fixed in phosphate-buffered 2% glutaraldehyde for two hours at room temperature, and post-fixed in 1% osmium in phosphate for one hour. Samples were washed with phosphate Sörensen buffer (TPS), dehydrated through a graded ethanol series and then embedded in Spurr’s resin (Spurr, 1969) [33]. Semi-thin (0.5–1 µm) and ultra-thin sections (500 nm) were obtained with a Reichert-Jung ultramicrotome (Ultracut E). Semi-thin sections were stained with toluidine blue (pH 9.0) for observation under a light microscope (LM) (Coolscope, Nikon). Ultra-thin sections were stained with uranium acetate and lead citrate as contrast agents and examined with a Jeol (JEM 2000 FX) transmission electron microscope (TEM).

### PCR Amplification, Cloning and Sequencing

DNA was extracted either from the gills of large individuals or from entire specimens using the QIAmp® DNA Micro Kit (Qiagen). For all specimens, a 579 bp fragment of the Cytochrome Oxydase I (COI) mitochondrial gene was amplified using primers LCOI 1490 [34] and H691 5′-GTRTTAAARTGRCGATCAAAAAT-3′ [30]. For selected individuals representing distinct COI haplotypes, a 999 bp fragment of the 28S rRNA nuclear gene corresponding to the domains D1, D2 and D3 was amplified using the primers C1’ (5′-ACCCGCTGAATTTAAGCAT-3′) and C4 (5′-TCGGAGGGAACCAGCTACTA-3′). PCR reactions were performed in a final volume of 25 µL containing approximately 3 ng of template DNA, 1.5 mM of MgCl_2_, 0.26 mM of each dNTP, 0.3 µM of each primer, 5% DMSO, and 0.75 unit of Taq polymerase (Qbiogene). Amplicons were generated by an initial denaturation step of 4 min at 94°C followed by 35 cycles of denaturation at 94°C for 40 sec, annealing at 50°C for COI and 52°C for 28S for 50 sec, and extension at 72°C for 1 min; and a final elongation at 72°C for 10 min.

The bacterial gene encoding 16S rRNA, used for the characterisation of bacterial communities, was amplified as described in Duperron et al. (2005) [35]. Symbiont diversity was evaluated for six specimens: two from the Philippines, two from Vanuatu (sunken wood) and two from New Caledonia (turtle bone). For each specimen, five PCR products were pooled and purified (Montage PCR kit, Millipore) prior to cloning using the QIAGEN PCR Cloning Kit. Inserts from a total of fifty positive clones were amplified using the plasmid-specific primers M13F and M13R.

PCR products were purified with AMPure XP beads, cycle-sequenced using the ABI BigDye Terminator v3.1 Cycle Sequencing Kit (1/32 reactions), and purified using an EtOH/sodium acetate precipitation. These products were then electrophoresed on an ABI PRISM (R) 3730×l Genetic Analyzer.

### Data Analysis

#### Genetic structure of mussel populations

Sequences were edited using Sequencher 4.1.4 and aligned using the Clustal W module implemented in Mega 4.0 [36]. Sequences were deposited both in BOLD and GenBank (COI: KC861674-KC861725; 28S: KC861726-KC861749 and KC904226-KC904234).

For the COI dataset, for each locality, the number of haplotypes (Hap), the number of segregating sites (Seg), haplotype (Hd) and nucleotide (Pi) diversity were determined using Arlequin 3.1.1.1 [37]. Phylogenetic relationships among haplotypes were constructed using the Median-Joining algorithm [38] and maximum parsimony post-processing calculation [39] implemented in Network 4.5.1.6 (http://www.fluxus-engineering.com).

F_ST_ was estimated among sampling locations using the formula of Weir and Cockerham (1984) [40] to assess population genetic structure. These calculations were based both on haplotype frequencies and on their pairwise nucleotide differences. A distribution of F_ST_ values under the null hypothesis of spatial genetic homogeneity was drawn from 10,000 datasets simulated by permutations of specimens between south-western localities. Genetic structure was also analysed with an exact test of differentiation using a Markov chain process with a burnin of 1000 steps. An Analysis of Molecular Variance (AMOVA; [41]) was performed among populations of the south-western part of the sampling region (Papua New Guinea, the Solomon Islands, Vanuatu and New Caledonia). An AMOVA was also assessed in order to evaluate hypothesised patterns of bathymetric genetic structure by using two depth categories (sampling<and >600 m). All tests were performed in Arlequin 3.1.1.1 [37].

Tajima’s D [42] and Fu’s FS [43] statistics were calculated to trace demographic events. Their statistical significance at alpha = 5% was estimated with a permutation test (10,000 replicates). Tests were performed for south-western localities, and for groups of localities among which no structure was detected. The parameters of a demographic expansion model, including the expansion factor (τ), the mutation parameter (θ = 2 µN, where µ is the mutation rate and N is the effective population size) initial (θ_0_) and actual (θ_1_) of the south-western populations were estimated using Arlequin. These estimators were used to plot the distribution of the number of pairwise differences between mitochondrial sequences (‘mismatch distributions’) based on Slatkin and Hudson (1991) [44] for south-western Pacific populations. Under the coalescent model modified from Hudson (1990) [45] 1,000 replicates were carried out to calculate parameters, to define 99% confidence intervals, and to calculate the sum of squared deviations (SSD) of these parameters between the observed and estimated mismatch distribution.

#### Phylogenetic relationships

Phylogenetic relationships at the intra-specific level were inferred from COI sequences. A sequence of the species *I. macdonaldi* (AY649804), which belongs to the same clade as *I. iwaotakii*, was added to the dataset as an outgroup. A phylogenetic tree based on the neighbour-joining (NJ) method of Kimura two-parameter (K2P) distances was built using MEGA version 4.0 [36]. The bootstrap analysis was based on 1000 replicates.

#### Symbiont diversity

The sequences obtained for the bacterial 16S fragment were aligned using the Clustal W module implemented by Mega 4.0 [36]. Sequences with ≥99% identity were considered as a single phylotype and a single representative clone from each phylotype was chosen for phylogenetic analysis. Sequences were deposited on GenBank (KC904235-KC904238). Sequences were compared with the NCBI (http://www.ncbi.nlm.nih.gov/) database using BLAST [46]. The most similar BLAST sequences were included in phylogenetic analysis. Phylogenetic reconstructions were performed using maximum likelihood (ML) and Bayesian (BA) analyses.

The most appropriate evolutionary model was estimated using jModelTest 0.1.1. The GTR+I+Γ model was chosen based on AIC. The ML analysis was performed using the software Treefinder [47] and node support was assessed with 1,000 bootstrap replicates [48]. BA was performed using MrBayes v3.1.2 [49]. Two independent analyses were run in parallel. Each analysis comprised four Markov chains and each chain was run over 8 million generations with a sampling frequency of one tree every hundred generations and a burnin period of 40,000 generations. Convergence between the two analyses was assessed using likelihood curves, standard deviation of split frequencies and potential scale reduction factor [50].

## Results

### Genetic Diversity and Phylogenetic Analyses

**Figure 3:**
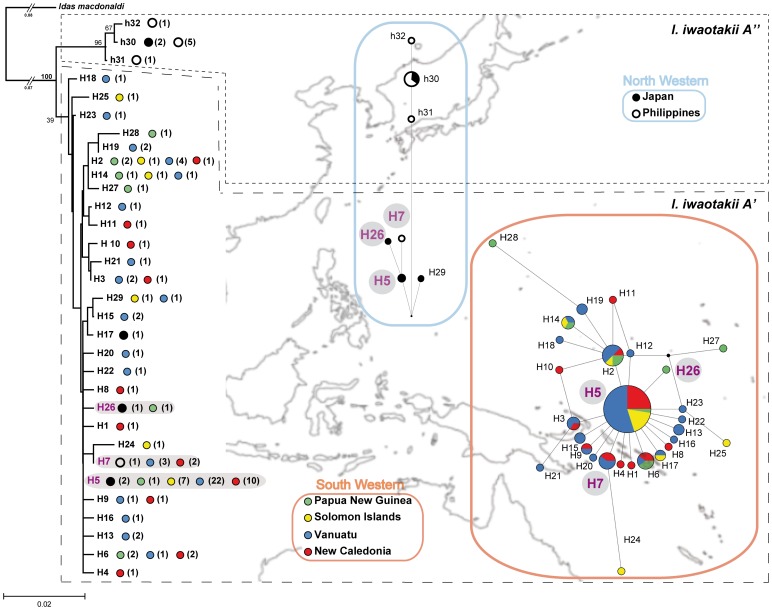
Population structure of *I. iwaotakii*. Left part: K2P neighbour-joining tree of unique COI haplotypes of *I. iwaotakii*. The localities at which each haplotype have been sampled is figured by a coloured circle (see legend for correspondence between localities and colours), the number of individuals sharing the same COI haplotype at each locality is given in brackets. The grey rectangles underline the haplotypes shared by the two studied regions. The scale bar represents 0.02% estimated base substitutions. The mitochondrial haplotypes of *I. iwaotakii* are divided in two clades, A’ and A’’, represented by one panel with large dots and the other one with small dots, respectively. Right part: Median-joining networks (MJN) from COI mtDNA data drawn independently for each of the two studied regions (North Western and South Western haplotypes). Size of haplotype circles is proportional to the number of specimens. Colours used to figure the localities within each region are the same of that used for the NJ tree. A small black circle represents a hypothetical haplotype. The haplotypes shared by the two studied regions are underlined using grey circles over haplotype labels.

A total of 50 COI mtDNA sequences of 579 bp were obtained. Moreover, 53 sequences available on Genbank were added to this new dataset for the analyses. Because the three sequences available on Genbank for Japan specimens are 430 bp (AB257520, AB257521 and AB257523) [51] and haplotype network reconstructions are sensitive to missing data, this analysis was performed on a reduced dataset of 430 bp long, in order to include as many specimens as possible. For each locality, the number of haplotypes (h), the haplotype (Hd) and nucleotide (π) diversities and the number of segregating sites (S) are given in Table 2. Indexes of diversity were low except for nucleotide diversity in populations from Japan, the Philippines and Papua New Guinea (4.467, 2.429 and 2.500 respectively). The neighbour-joining (NJ) tree constructed from unique COI haplotypes splits the specimens into the two clusters detected in previous studies [7], [30]. The first (A’’) includes specimens from the north-western localities only (two specimens from Japan and seven from the Philippines), and the second (A’) pools all specimens from the south-western localities (Papua New Guinea, the Solomon Islands, Vanuatu and New Caledonia) but also four specimens from Japan and one from the Philippines (Fig. 3).

**Table 2:** Geographical location, number of sequences (N), number of haplotypes (h), haplotype diversity (Hd) (with standard deviation), nucleotide diversity (π) and the number of segregating sites (S) of *I. iwaotakii* populations.

Localities	N	h	Hd (SD)	π	S
Japan	6	4	0.867 (0.129)	4.467	9
Philippines	8	4	0.643 (0.184)	2.429	9
PNG	9	7	0.9444 (0.070)	2.500	9
Solomon Islands	12	6	0.682 (0.149)	1.47	8
PNG+Solomon Islands	21	10	0.8429 (0.069)	1.962	14
Big−Bay	14	9	0.8791 (0.079)	1.38	8
Malo	24	12	0.7572 (0.094)	1.275	11
Malekula	10	5	0.7556 (0.130)	0.956	4
Vanuatu total	48	19	0.8023 (0.058)	1.268	18
New Caledonia	22	11	0.797 (0.087)	1.221	9

PNG = Papua New Guinea.

### Connectivity Patterns

Two separate median-joining networks were computed for the north-western and south-western regions, as these locations harboured distinct phylogenetic lineages and the sampling effort was very unbalanced between the two regions. In agreement with the NJ analysis, median-joining networks revealed the co-occurrence of both lineages in the Northwest (Fig. 3). The south-western median-joining network displayed a ‘star-like’ topology with one central and common/ancestral haplotype surrounded by several rare, derived haplotypes. Two 28S haplotypes, separated by one substitution, were obtained from 34 specimens. The first haplotype was represented by 25 specimens from the south-western area (lineage A’), two from Japan (one specimen from lineage A’ and one from lineage A’’) whereas the second haplotype was represented by seven specimens from the Philippines (lineage A’’). The Northwest (Japan and the Philippines) and the south-west areas (other localities) were significantly differentiated (FST = 0.61485, p-value <0.001). FST tests were not significant among localities within each of these two areas. The only significant exact tests (results not shown) were obtained when comparing the Philippines sample with the south-west localities. The non significance of the exact tests performed between Japan and any other locality should be interpreted with caution given the small sample size and the co-occurrence of the two lineages in Japan. No significant population structure was detected among south-western localities (AMOVA results - geography: FST = −0.00540, p-value = 0.7; bathymetry FST = −0.00398, p-value = 0.6; Table 3).

**Table 3:** AMOVA results comparing genetic variation in *I. iwaotakii* populations at two hierarchical levels.

	Source of variation	Degrees of freedom	Variance components	FST	P values
Geography	Among populations	2	−0.00376	−0.00540	0.66020
	Within populations	86	0.70109		
Bathymetry	Among populations	1	−0.00292	−0.00398	0.61762
	Within populations	87	0.73635		

Populations are clustered according to geographical locations (among populations from Papua New Guinea and the Solomon Islands grouped together, Vanuatu and New Caledonia) and depth ranging, below and above 600 m, from samples collected in the South-West of the Pacific Ocean.

### Demographic Events

**Figure 4:**
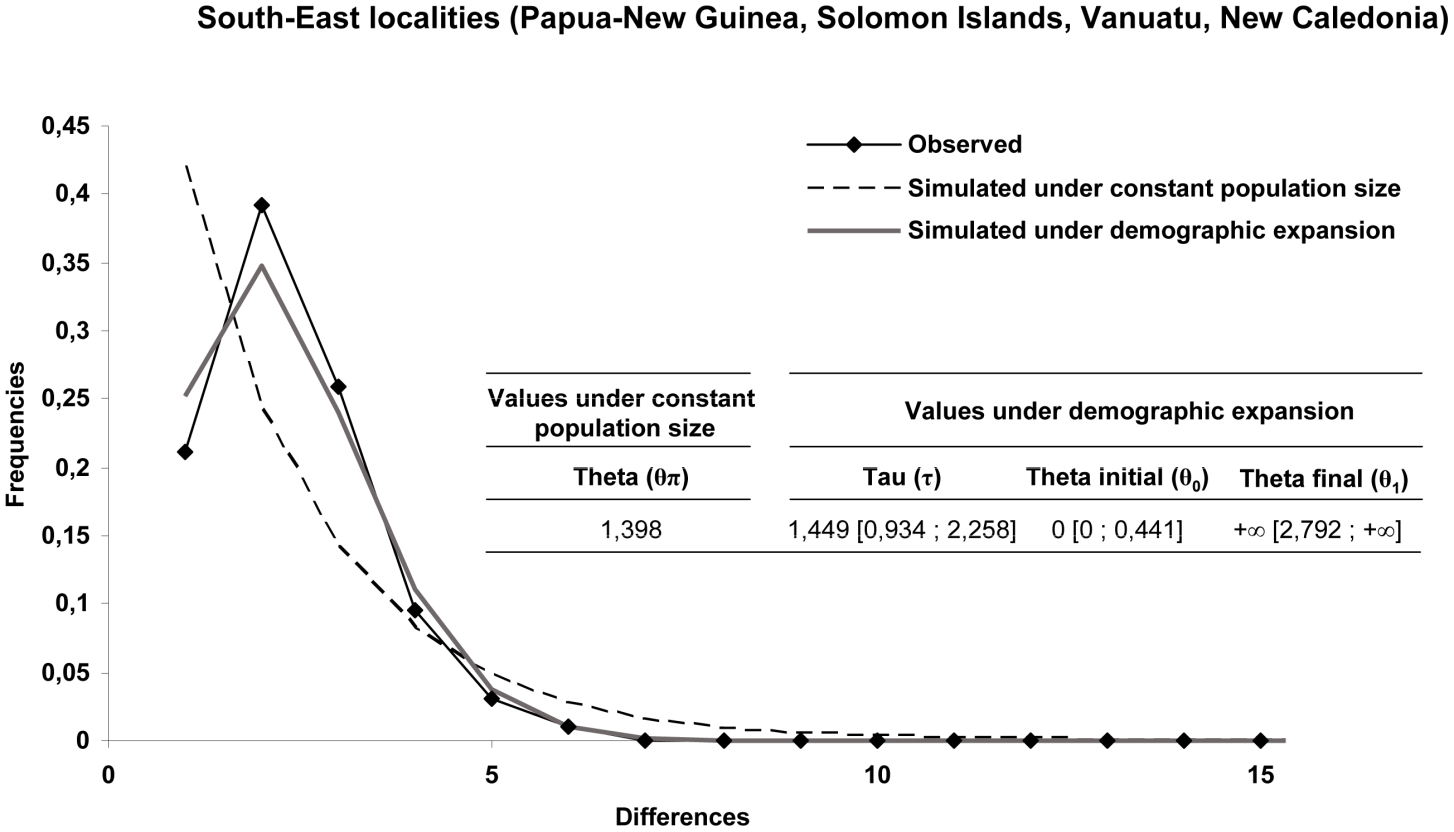
Mismatch distributions of COI haplotypes. COI haplotypes observed, simulated under a constant population size and a model of population expansion for the South-west Pacific populations of *A. iwaotakii*. Along x-axis are indicated the numbers of nucleotide differences between all pairs of sequences and on y-axis the frequencies of pairs. The table summarizes the parameters of demographic analyses under constant population size and demographic expansion. The confidence intervals at 99% of the parameters under a demographic expansion model are given in square brackets.

Tajima’s D and Fu’ FS statistics were negative for each of the south-west localities (except D for Papua New Guinea and Malekula), pointing to significant evidence of population expansion (Table 4). When grouping localities according to the genetic structure, both tests remained significantly negative for the south-west. Observed mismatch distributions were unimodal and not statistically different from the simulated distribution under an expansion model (Fig. 4).

**Table 4:** Tajima’s D-statistics and Fu’s FS estimated for each location of the south-western part of the sampling plan and for genetically homogeneous subgroups.

Localities	N	Tajima's D	Fu's Fs	Tajima's D	Fu's Fs
PNG	7	−0.80987	−2.43623*	−2.23757***	−28.65131***
SolomonIslands	12	−1,77787*	−2.17434*		
PNG+SolomonIslands	19	−1.88971*	−6.49069***		
Big−Bay	14	−1.69536*	−6.65887***		
Malo	24	−1.92048**	−9.86651***		
Malekula	10	−1.24468	−2.37682**		
Vanuatu total	49	−2.17746**	−19.45298***		
New Caledonia	22	−1.69443*	−8.74973***		

Significant values are noted * (0.01<p≤0.05), ** (0.001<p≤0.01) and *** (p≤0.001). PNG = Papua New Guinea.

### Larval Shell Morphology

**Figure 5:**
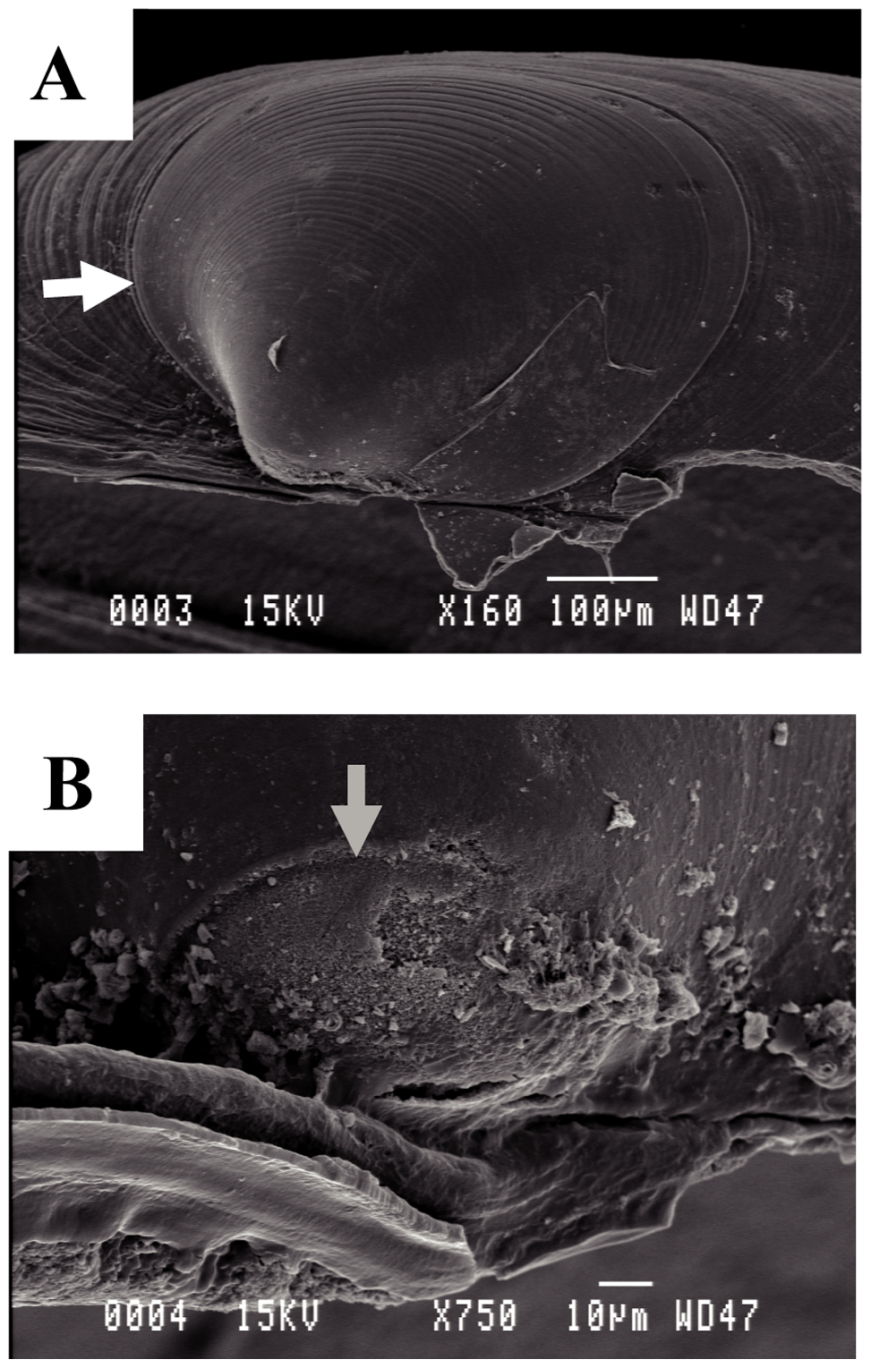
Scanning electron micrographs of the larval shell of a specimen of *Idas iwaotakii*. Pictures: (A) Dorsal view showing prodissoconch I (PI) and prodissoconch II (PII); the white arrow indicates the boundary between the dissoconch and prodissoconch, (B) Detail of PI; the grey arrow indicated of the boundary between PI and PII.

Larval shells (prodissoconchs) were 544.33 µm (SD: ±37.58) in diameter, had a reddish colour and were easily distinguishable from the yellowish post-larval shell (dissoconch). The prodissoconchs II displayed numerous concentric lines (Fig. 5A); whereas the prodissoconchs I, situated close to the umbo, had a granulated texture and were 74.05 µm (SD: ±9.35) in diameter (Fig. 5B). The size of the prodissoconchs I, which corresponds to the early stages of veliger larvae, is directly related to that of the oocytes. The small size of the prodissoconchs I compared to the prodissoconchs II, suggested the absence of a yolky egg. Prodissoconch II records the duration of pelagic larval life. Thus, the well-developed prodissoconchs II observed here suggested that larvae spend a significant part of their life in the plankton. The few available data on related species suggested that these larvae could have dispersed over hundreds of kilometres [23].

### Symbiont Diversity and Phylogeny Based on 16S rRNA Gene Sequence Analysis

The bacterial 16S rRNA alignment was 1459 bp long, and included 50 sequences corresponding to 9 phylotypes. A GenBank BLAST analysis revealed that phylotypes 1, 2, 3 and 4 were >97% similar to thiotrophic symbionts of bathymodioline mussels. Among those, phylotype 1 was dominant, represented by 35 clones from all analysed specimens (i.e. independently of locality and substrate). Phylotypes 2 and 3, each represented by 2 clones, were recovered from two specimens from the Philippines. Phylotype 4, including 6 clones, was recovered from only one specimen from the Solomon Islands and one from New Caledonia.

The five other phylotypes were singletons and bear no resemblance with mussel symbionts (results not shown). They most likely represent environmental bacteria, as they were >97% similar to Proteobacteria (epsilon- and gamma-Proteobacteria), Planctomycetes, and Bacteroidetes (*Cytophaga Flavobacter Bacteroides*, or CFB) that are also commonly found at/near vent communities.

**Figure 6:**
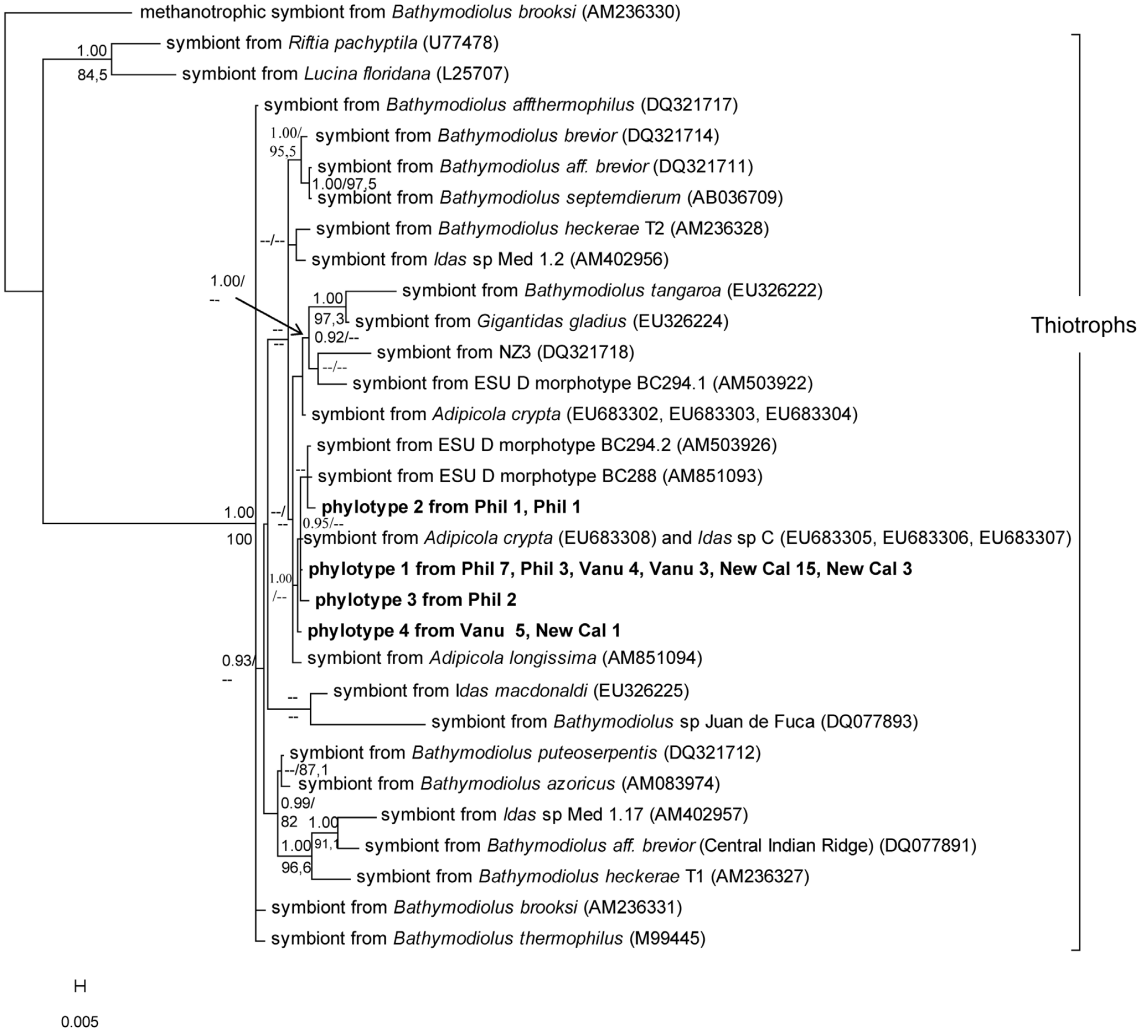
Bayesian tree displaying bacterial symbionts based on the analysis of the 16S rRNA. Phylotypes associated with *I. iwaotakii* are shown in bold. Posterior probabilities (PP) and bootstrap values obtained from ML analysis are given above and below branches respectively. PP and bootstrap values lower than 0.90 and 50%, respectively, are not shown. The scale bar represents 0.5% estimated base substitution.

Only thiotrophic symbiont sequences (corresponding to phylotypes 1 to 4) were included in the phylogenetic analyses. Trees were rooted on the methanotrophic symbiont of *B. brooksi* [30], [52]. The topologies of the ML and BA reconstructions were identical, and confirmed that phylotypes 1 through 4 belonged to a single large clade which included all known thiotrophic symbionts of the Bathymodiolinae and other mussels from sunken organic substrates (Fig. 6). Moreover, these four phylotypes are resolved within a group of symbionts of organic falls mussels. The four phylotypes were closely related to symbionts of tiny mussels associated with sunken wood from the Philippines (AM503926, AM851093) [21] and to the symbionts of *G. crypta* and *I.* sp. C from Vanuatu [30].

### Localisation of Associated Bacteria

**Figure 7:**
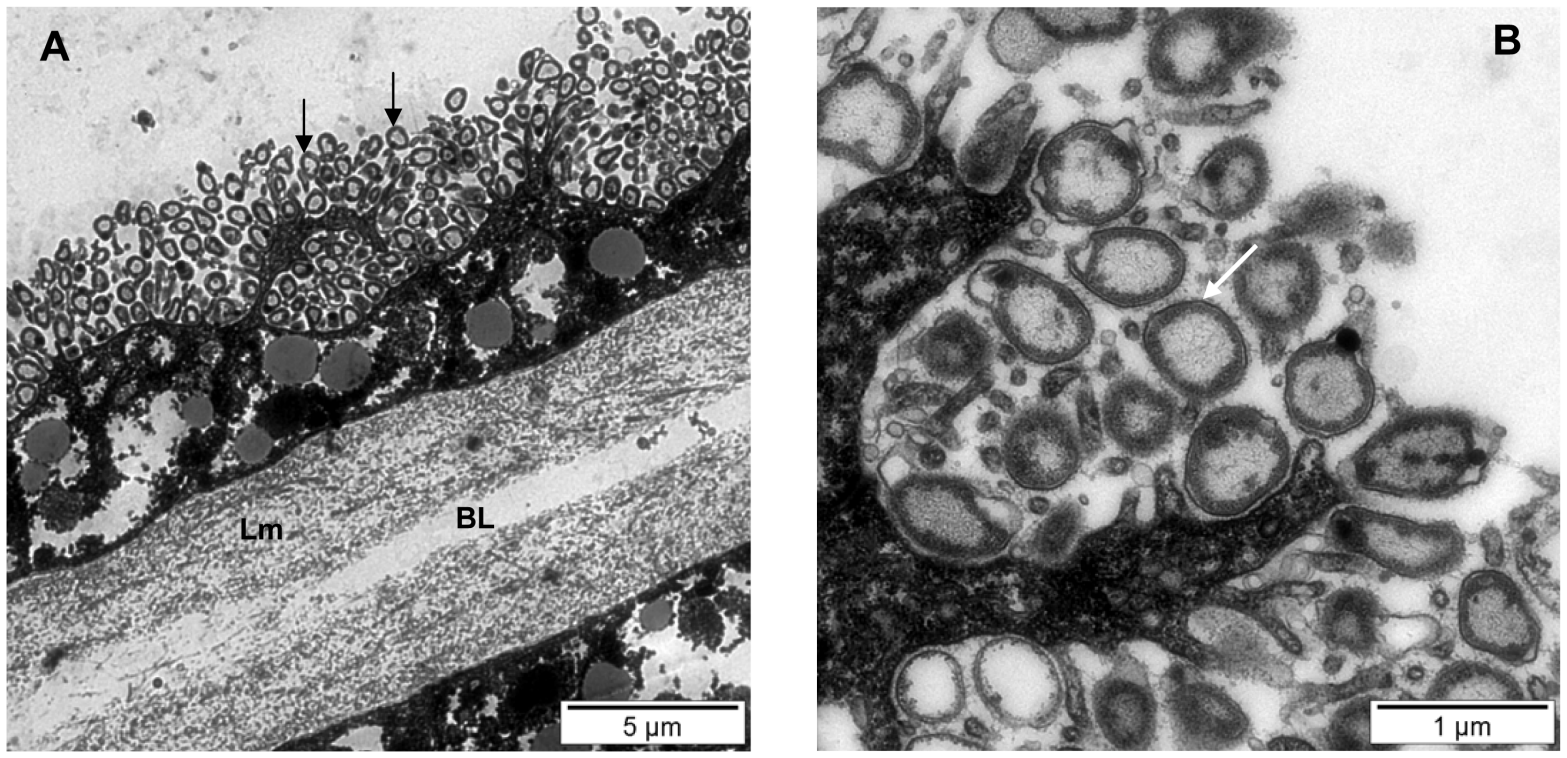
Gill filaments of *Idas iwaotakii*. (A) TEM view of gill filament of the lateral zone from a specimen of *I. iwaotakii* collected on wood off Vanuatu. Bacteria (black arrows) are located extracellularly in contact with microvilli. BL: blood lacuna; Lm: basal lamina. (B) Electron micrograph of the extracellular symbionts. Symbiotic bacteria possess a double membrane (white arrow) typical of Gram negative bacteria.

TEM observations of sections of gill filaments indicated the presence of extracellular, gram negative bacteria located between the microvilli of the host cells, throughout the lateral zone of each gill filament (Figs 7A–B).

### Digestive Tract Structure

**Figure 8:**
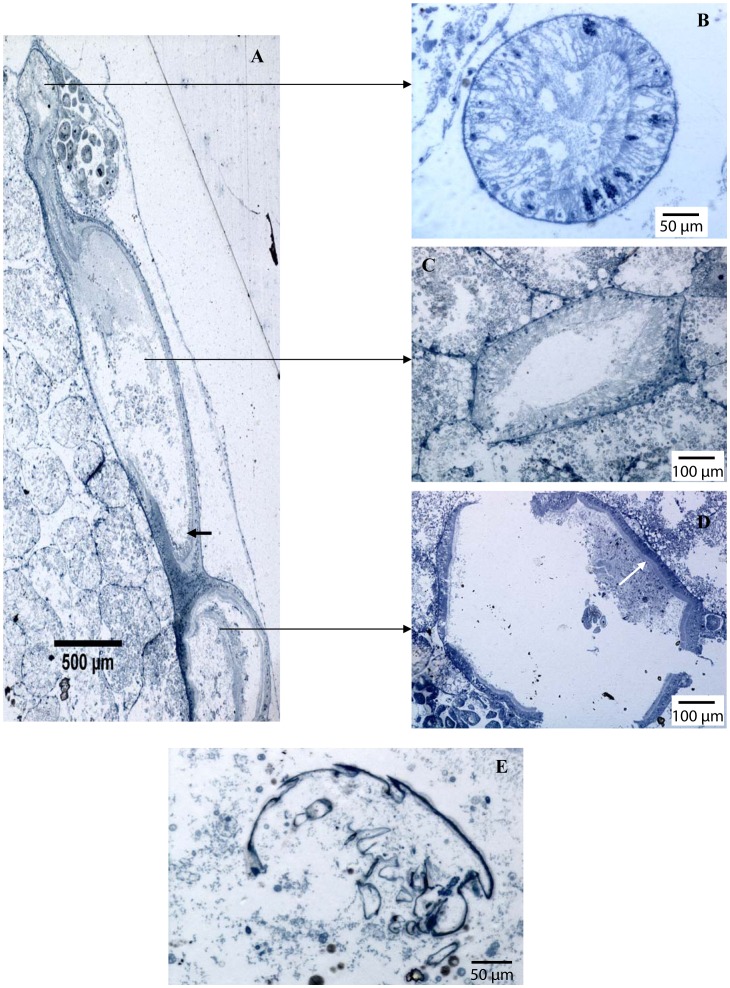
Semi-thin sections of the digestive tract of *Idas iwaotakii*. The specimen was collected on turtle bone off New Caledonia. (A) Overall view of the digestive tract (longitudinal sections). The black arrow indicates the location of microvilli in the intestine. (B) Cross section of the oesophagus lined by a stratified epithelium. (C) Cross section of the intestine. (D) Cross section of the stomach lined by a cuboidal epithelium. The white arrow indicates the gastric shield. (E) Longitudinal section of a detail of intestinal contents.

LM observations of semi-thin sections of the digestive system revealed the presence of typical features of bivalve digestive tract consisting of an oesophagus, a stomach, an intestine and a well-differentiated digestive gland that surrounds the stomach and a part of the intestine. In all observed specimens: the unilaminar prismatic epithelium of the oesophagus had ciliated cells (Fig. 8B); the stomach epithelium had cubic cells (Fig. 8D) and a chitinous area corresponding to the gastric shield; the prismatic epithelium of the intestine (Fig. 8C) displayed a high density of microvilli. The digestive tract was curved and formed at least one loop (Fig. 8A). Both LM and TEM observations revealed the presence of digestive contents in the stomach and the intestine. The alimentary bolus was nevertheless difficult to identify. One rigid structure, about 296 µm wide and little damaged by digestion, could be identified as a foraminiferan test (Fig. 8E).

## Discussion

### Genetic Divergence, Population Connectivity, and Demographic History

Our study confirms that *Idas iwaotakii* populations are divided into two distinct mitochondrial lineages, A’ and A’’, and confirmed their coexistence in Japan. But, although we confirm that specimens from the lineage A’’ from the Philippines have a distinct 28S rRNA haplotype, we show that in Japan, specimens from both A’ and A” lineages share the 28S rRNA haplotype typically associated with A’ in the south-western area. The combination of nuclear and mitochondrial data on a larger number of mussels thus suggests that the two mitochondrial lineages are not, as it has been hypothesised before [7], reproductively isolated.

At the regional scale (SW Pacific) the sampling of *I. iwaotakii* initiated by Lorion et al. (2009, 2010) [7], [30] is improved here. Although the localities are separated by 500 km (New Caledonia/Vanuatu) to 2400 km (New Caledonia/Papua New Guinea), no genetic structure was detected. Moreover, our sampling effort covers a wide depth range, and the recruitment experiments deployed off New Caledonia allowed us to collect *I. iwaotakii* on bone, a substrate much more difficult to locate than sunken wood. Indeed, trees and other plant substrates can reliably be found in the vicinity of islands and continents skirted with well-developed coastal forests, whereas the distribution of skeletal remains has an unpredictable, idiosyncratic distribution. The New Caledonian specimens sampled from turtle bones show no genetic difference from other samples collected from sunken-wood at other localities. Similarly, no genetic structure is detected over a large bathymetric range (i.e. 460 m –2,307 m). Thus, *I. iwaotakii* is a widespread species that may colonise, over a wide depth range, organic substrates such as sunken-wood and bone [7]. These results are congruent with those available for other species associated to organic remains (i.e. *I.* sp. C and *G. crypta* [30]).

The absence of genetic structure between south-western sampling localities suggests either high larval dispersal ability and an absence of barriers to dispersal or historical connectivity still detectable with COI and 28S. These results are consistent with the long larval stage here inferred from the size of prodissoconch II, and documented for close relatives from hydrothermal vent and cold seep environments. Indeed, larvae from *B. azoricus* could remain in the water column for 5 to 6 months [53], [54]. Evidence of long-distance dispersal has also been found for *I. modiolaeformis*, *I. argenteus* and other Bathymodiolinae [14], [17], [23], [55]–[58]. Notably, Lutz et al. (1980) [23] revealed that *B. thermophilus* produces large numbers of small eggs that develop into planktotrophic larvae and potentially disperse over long distances. The assumption of large-scale dispersal ability is supported by the high gene flow documented among *B. thermophilus* populations from 13°N to 11°S latitude on the East Pacific Rise (∼ 2,500 km) [24], [26]. Vents, seeps and organic remains, are patchy and often ephemeral habitats. In such highly fragmented habitats, establishment and maintenance of populations probably relies on the long, free-swimming, larval stage that in turn might also explain the wide geographical distribution of most species.

Based on our genetic data, we hypothesise that a small number of migrants from the northwest area have recently founded new successful populations in the south-western area. The association of a demographic expansion signal and a reduction of the genetic diversity in the southern populations support this hypothesis. However, this pattern is also consistent with allopatric divergence followed by secondary contact. The complex history of the Western Pacific, marked by tectonic plate movement (e.g. [59], [60]), volcanism and periodic sea level changes (e.g. [61]), might have impeded larval dispersal, during some periods, and thus favoured the emergence of new lineages through founder events but also the secondary contacts among isolated populations.

Geographic breaks between pairs of closely related species have been repeatedly documented in both *Bathymodiolus s.s* and *Idas* lineages. In *Idas*, the cold seep species *I. modiolaeformis* and *I. macdonaldi* (3.3% divergent at COI), were proposed as a potential amphi-Atlantic species-complex [17]. In the *Bathymodiolus* lineage, the same was proposed for the seep complex of species *B. boomerang* and *B. aff boomerang* (2% divergent at COI, [62], [63]). Two pairs of vent species have a geographic structure along a mid-oceanic ridge system. The two sister-species *B. thermophilus* and *B. antarcticus* are respectively distributed in the Galapagos rift to the North and not to the South of the East Pacific Rise [26], [27], [64]. Similarly the sister-species *B. azoricus* and *B. puteoserpentis* are respectively distributed in the North and in the South part of the Mid Atlantic Ridge [65], [66]. However, in the Indo-West Pacific, the populations attributed to *B. septemdierum*, *B. brevior* and *B. marisindicus*, are not reproductively isolated and display no genetic structure over a very wide geographical area (see [5]).

### Symbiotic Association and Insights into Nutritional Features of I. iwaotakii

In previous studies [21], [30], [67], thioautotrophic symbionts have predictably been found in each newly examined deep-sea mussel associated with organic remains. This new dataset supports this predictability and moreover shows that, within a species, the same dominant bacterial phylotype is found, independent of geographic location (the Philippines, Vanuatu and New Caledonia), substrate type (wood versus bone) or depth (441 m to 1767 m). This dominant phylotype is closely related to a symbiont detected in another species within the *Idas* lineage (*I.* sp. C) and also in another species from a distinct lineage (*G. crypta*) [30]. These two species were also collected both on wood and bone. Phylogenetic relationships among sulphur-oxidizing symbionts are however poorly resolved. This lack of resolution is probably due to the relatively slow evolution rate of the 16S rRNA. More variable genetic markers have to be developed to better resolve symbiont phylogeny.

Electron microscopic analyses confirm the presence and abundance of bacteria in association with gill filaments. The bacteria are extracellular and are thus directly exposed to the reduced compounds from decaying organic remains. The extracellular localisation of symbionts has already been documented for other mussels associated with wood falls, notably for the *Idas* lineage in *I.* sp. C and *I.* sp. D [21], [30], [67], [68]. By contrast, the symbionts of bathymodioline mussels from hydrothermal vents and cold seeps are mostly intracellular. Likewise, *I. washingtonius*, which is associated with whale bone, possesses intracellular bacteria located within gill bacteriocytes [18], [30]. Thus, by contrast with the species included in *Bathymodiolus s.s.* lineage, the species included in the *Idas* lineage harbour either intracellular symbionts (*I. macdonaldi* or *I. washingtonius*) [18] or extracellular symbionts (*I. iwaotakii*, *I.* sp. C and *I.* sp. D) [8], [30]. As *I. washingtonius* is sampled on organic remains, we suggest that the extra- or intra-cellular location of the bacteria is not strictly related to the environments as was previously suggested [5], [6].

Bathymodioline mussels possess a functional but considerably reduced digestive tract [11]–[14]. Some *Bathymodiolus* species (*B. boomerang*, *B. heckerae*, *B. brooksi*, *B. thermophilus* and *B. aff. thermophilus*) have a simple digestive tract with a small stomach and a straight midgut without any coiling or loop [14], [69], [70]. Others such as *B. azoricus* and *B. puteoserpentis* have a coiled intestine and well-developed labial palps [71], [72]. Intestines with recurrent loops have been observed in seeps specimens of *I. macdonaldi* [14]. Based on stable isotope measurements, Deming et al. [18] suggested that *I. washingtonius*, may have a mixotrophic nutritional strategy involving both filter feeding and supply from symbionts. *I. iwaotakii* has a coiled intestine. Digestive contents were relatively abundant and varied. For example a rigid structure found in the intestine could be a foraminiferan test resembling *Spiroplectammina taiwanica* (Chang, 1956) ([73]; see figures 29 A and B). Le Pennec and Prieur (1984) [11] also identified the tests of benthic Foraminifera and diatom frustules in the stomach contents of vent mytilids.

Overall, sulphur-oxidizing symbionts closely related to those previously identified in Bathymodiolinae are present in the gills of *I. iwaotakii* and the digestive tract is non-reduced and functional. These mussels associated with organic falls are thus potentially able to obtain carbon and other nutrients either from their autotrophic symbionts and/or by filter feeding. A longer intestine with recurrent loops suggests a significant contribution of filter feeding to the mussels’ diet, likely supplemented by nutrition derived from symbiont chemoautotrophy (e.g. [70]). The relative contribution of each pathway remains to be quantified. The concentration of hydrogen sulphide (H_2_S) available in the environment determines the ability of bacterial symbionts to provide food supply. Concentrations of reduced compounds probably vary depending on quantity, nature and state of degradation of the available organic substrates (e.g. [74]). A mixed diet might allow individuals to adapt to high variability in the availability of reduced compounds.

### Evolutionary Significance of Biological Features Related to Environments

**Figure 9:**
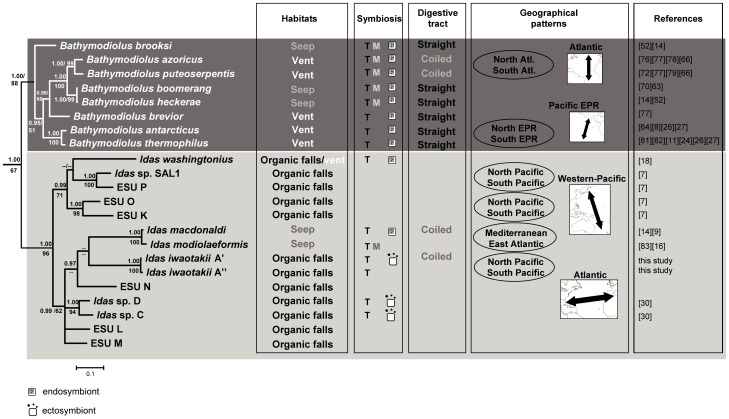
Comparison of data available for the *“thermophilus”* lineage and its sister lineage. The “*thermophilus*” lineage is represented by the dark grey rectangle and its sister lineage, corresponding to the genus *Idas*, by the light grey rectangle. Phylogenetic relationships correspond to a part of the combined COI mtDNA and 28S RNA phylogenetic tree from [7]. T: thiotrophic symbiont; M: methanotrophic symbiont. In geographical patterns section, the ovals correspond to pairs of sister-species with an allopatric distribution and location maps illustrate the allopatric distribution of these sister pairs. References: [7]–[9], [11], [14], [16], [24], [26]–[27], [30], [52], [63]–[64], [66], [70], [72], [76]–[83].

Although living on organic remains, *Idas iwaotakii* shares many biological similarities with the large mussels of *Bathymodiolus s.s* living in either vents or seeps: (i) association with chemoautotrophic symbionts, (ii) high larval dispersal and (iii) lack of geographical genetic structure. However, *I. iwaotakii* apparently accommodates to a wider range of ecological conditions than *Bathymodiolus s.s.* species (Fig. 9). Indeed, *I. iwaotakii* displays a very wide geographical distribution and a large bathymetrical range, has probably a mixed diet and is able to colonise various organic substrates (i.e. bone and vegetal remains). By contrast, *Bathymodiolus s.s.* species are restricted either to vents or seeps but are never found in both habitats nor on organic remains. This contrasts with giant mussels belonging to the *“childressi”* group that, like *Bathymodiolus* (sensus lato) *platifrons* or *B.* (s.l.) *japonicus* are found both at vent and seep sites [75]. The straight intestine evidenced for most *Bathymodiolus s.s.* species suggests a greater dependency on the food supply provided by their autotrophic symbionts. This dependency may explain why they are restricted to habitat in which the reduced compounds remain available regularly, even if at variable concentrations.
